# Supplementary figures and images for: Vector genetics, insecticide resistance and gene drives: An agent-based modeling approach to evaluate malaria transmission and elimination
Source: PLoS Comput Biol. 2020 Aug 14;16(8):e1008121. doi: 10.1371/journal.pcbi.1008121 (PMC7449459; doi:10.1371/journal.pcbi.1008121)

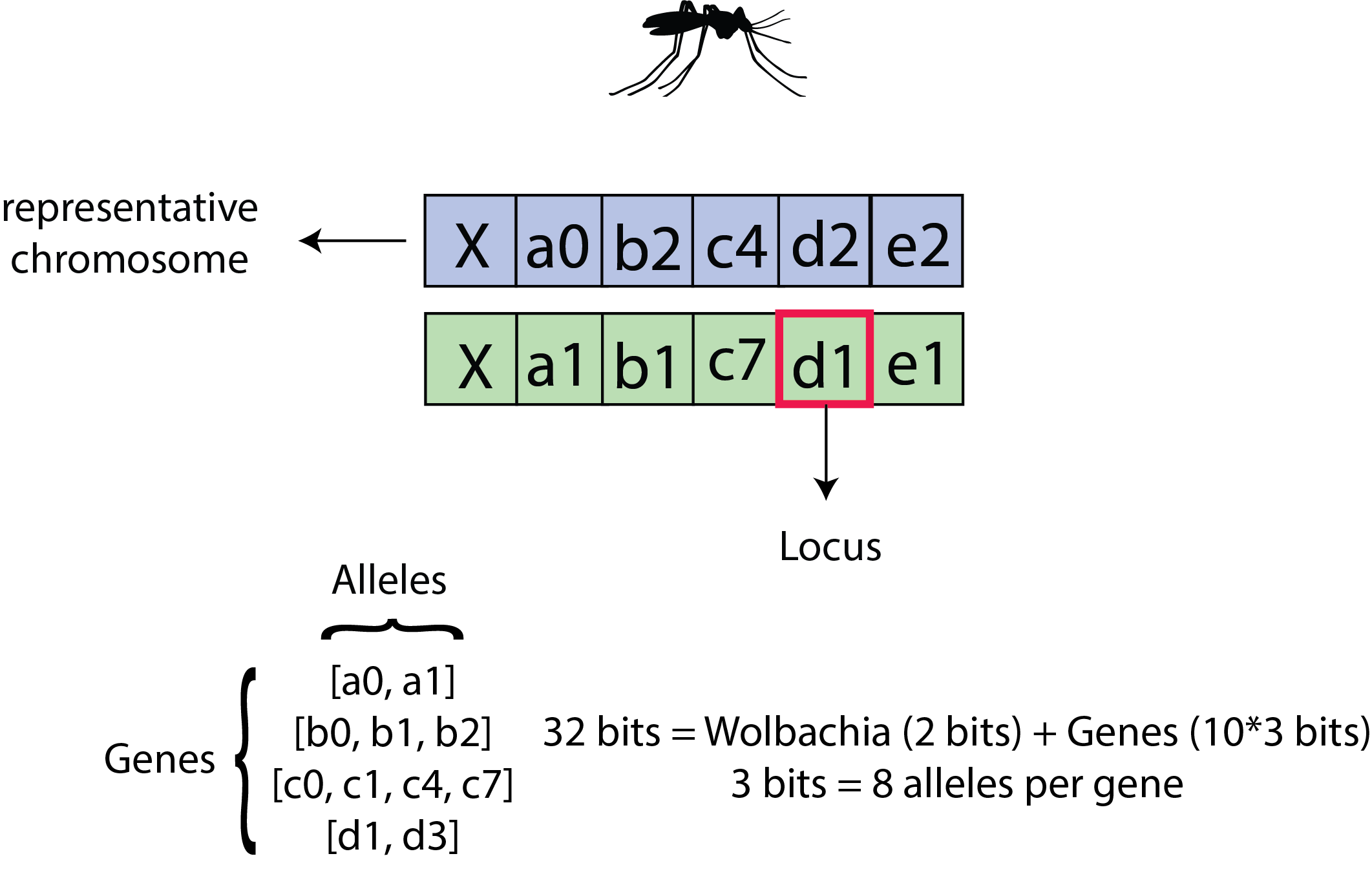

Supplement: S1 Fig — Each vector or vector cohort carries with it 64 bits of memory dedicated to a diploid carrying 10 different representative genes with up to 8 different alleles per gene. 4 bits are reserved for microbial interventions such as Wolbachia, metrhizium or microsporidia. While a mosquito has 3 chromosome, the representative genome here consists of only one pair of representative chromosomes. (TIF) [file pcbi.1008121.s001.tif]

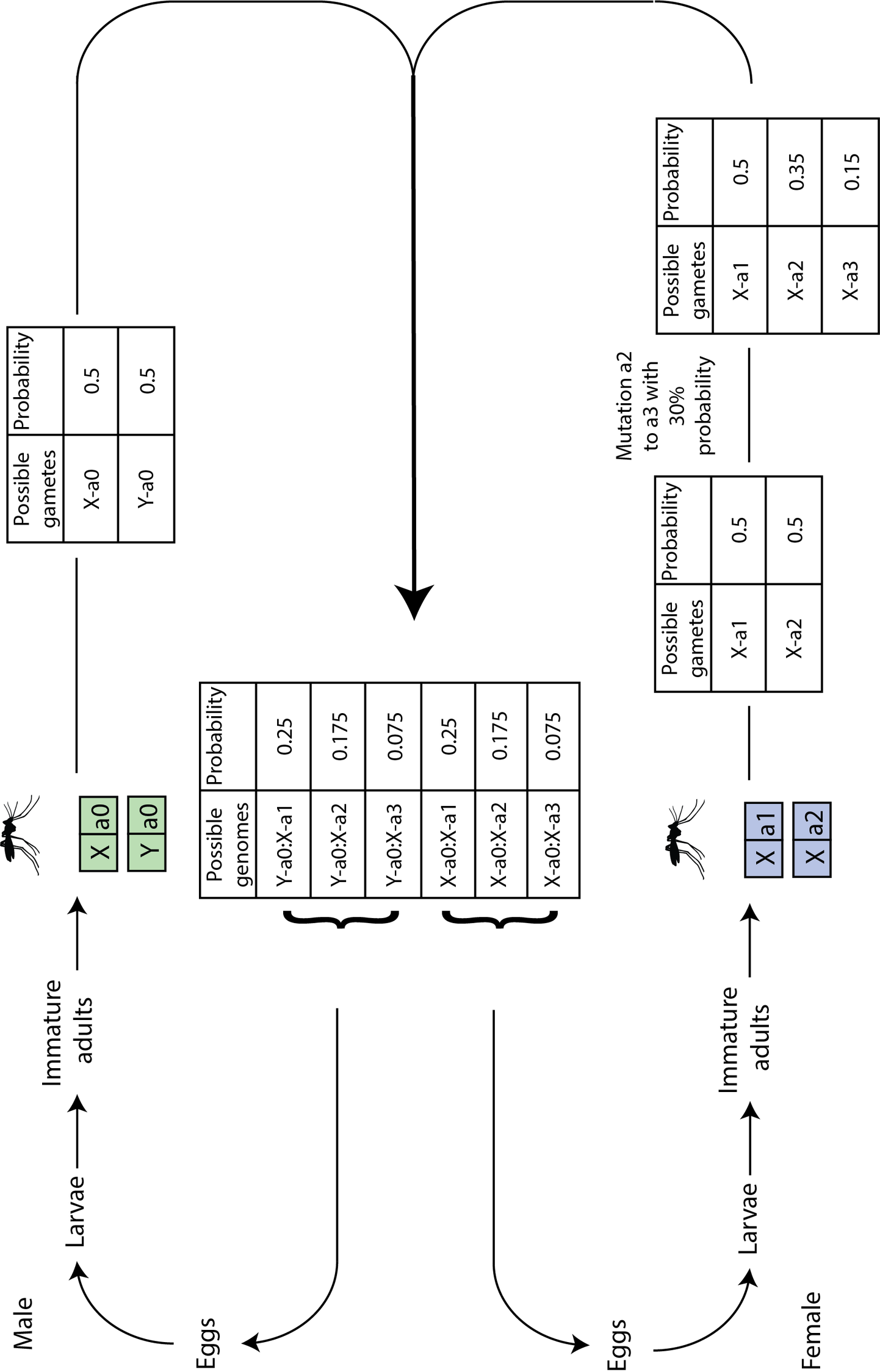

Supplement: S2 Fig — EMOD adopts a 4 part lifecycle for the vector starting from eggs that progress to the larval stage before moving onto the immature adult and adult stage. When adults mate, genomes from male and female mosquitoes are used to calculate the likelihood of existence of a gamete carrying a certains set of allele combinations. Random mutation are then applied and possible genome probabilities calculated. Mutation and recombination rates used here are illustrative and do not reflect a specific real world phenomenon. These probabilites are multiplied by the egg batch size, which is modeled as a phenotypic property, for each species to obtain the number of eggs bearing each genome. (TIF) [file pcbi.1008121.s002.tif]

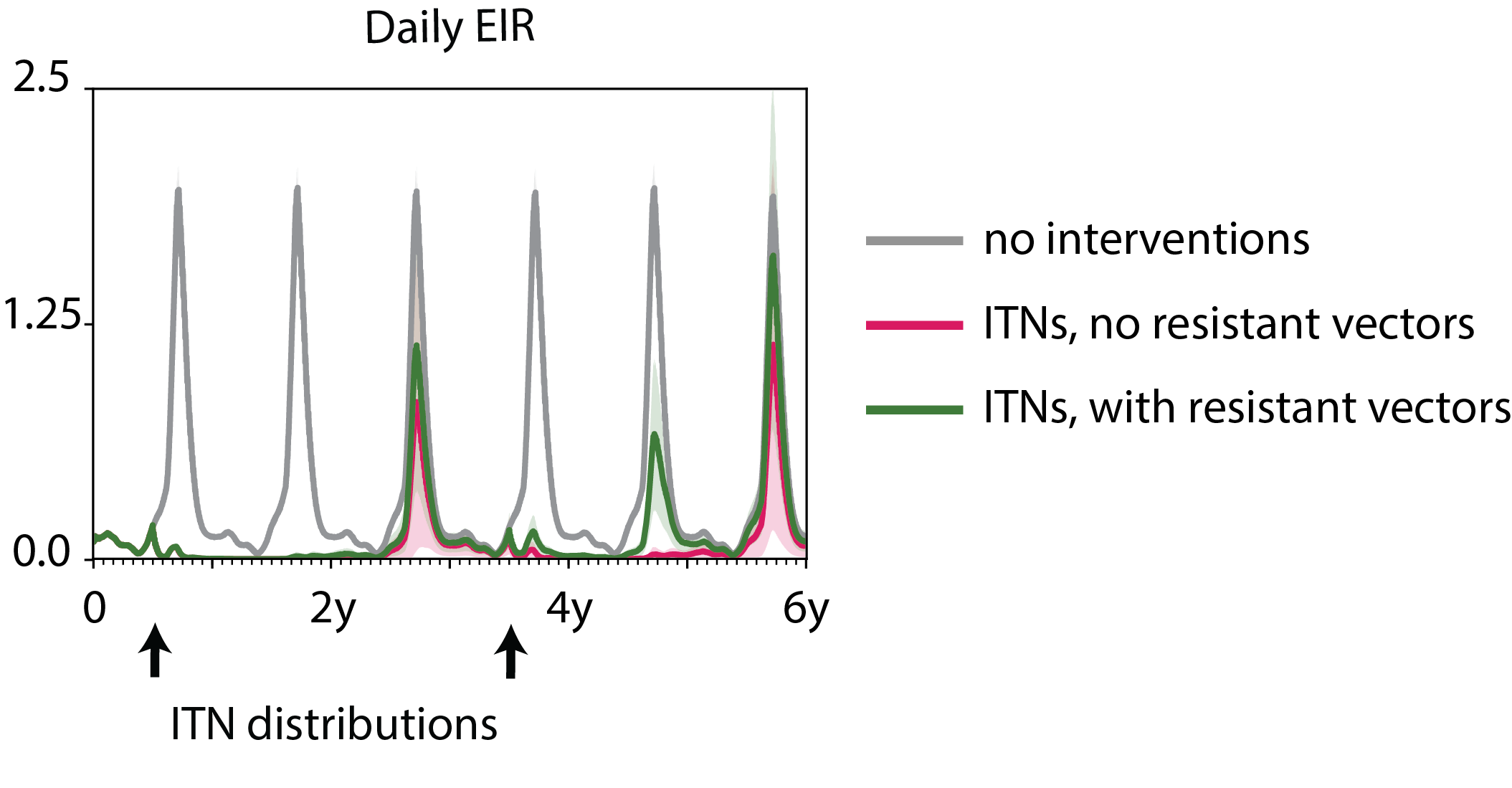

Supplement: S3 Fig — The shaded area around the mean represents one standard deviation calculated across the 50 stochastic realizations. ITNs are distributed every three years at the beginning of the peak season. (TIF) [file pcbi.1008121.s003.tif]

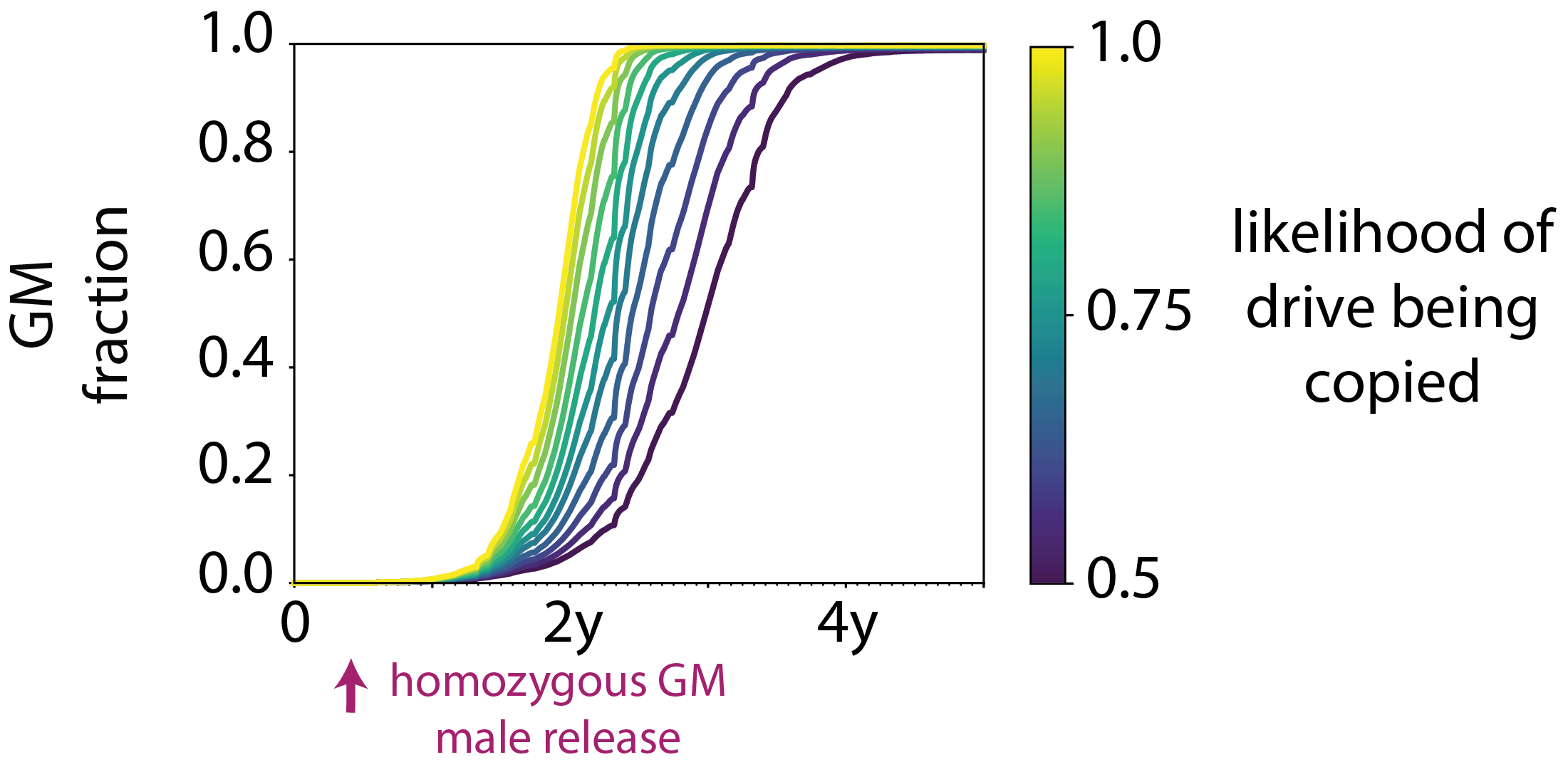

Supplement: S4 Fig — There are no other vector control interventions. And transmission to human is set to 0. (TIF) [file pcbi.1008121.s004.tif]

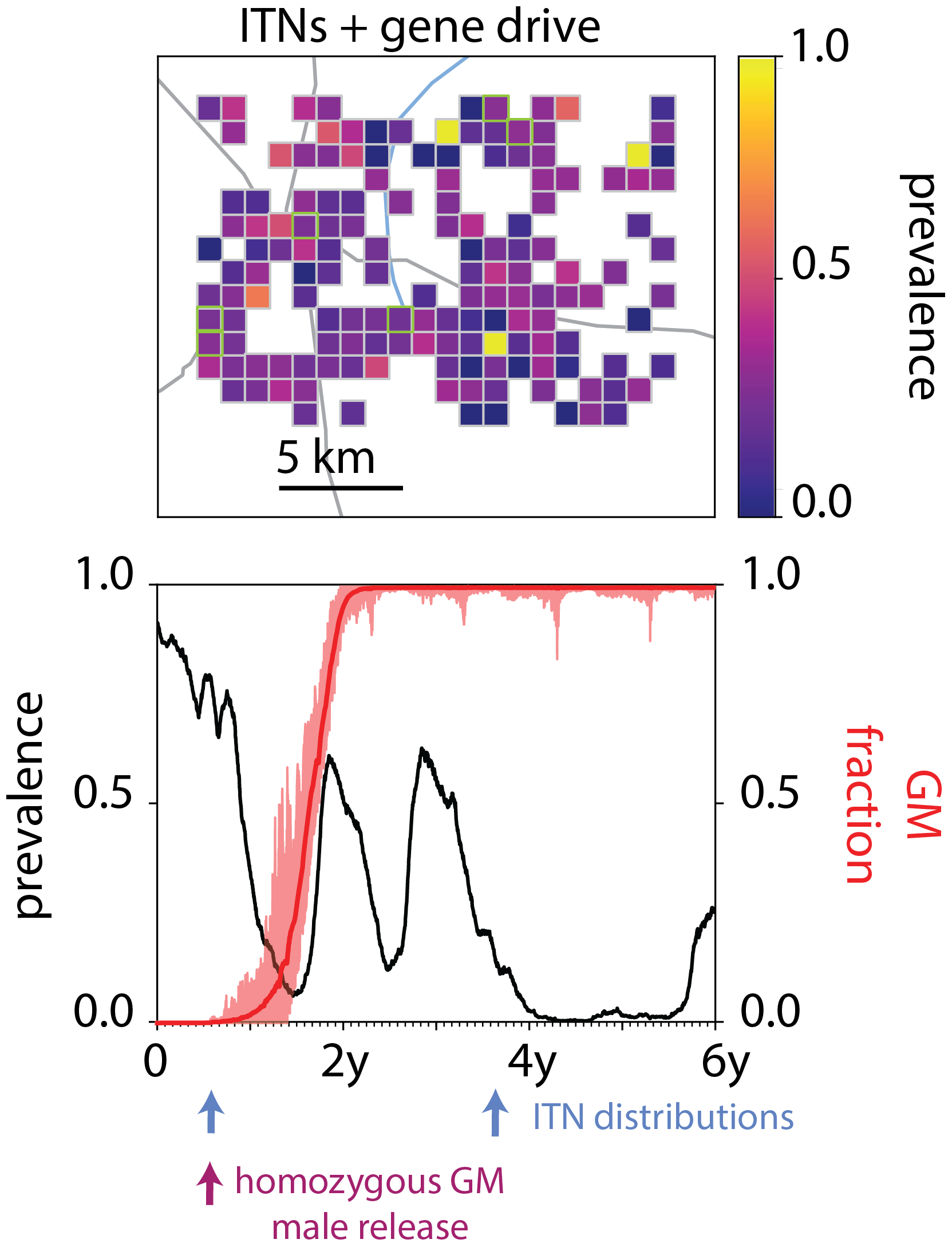

Supplement: S5 Fig — Upper panel describes spatial distribution of prevalence at the end of six years. Blue line in lower panel describes total prevalence in simulated area. Black line describes overall establishment of GM mosquitoes over time while the red envelope describes the range of establishment rates across all spatial nodes. (TIF) [file pcbi.1008121.s005.tif]
